# Genome-wide profiling of unmodified DNA using methyltransferase-directed tagging and enrichment

**DOI:** 10.1016/j.crmeth.2025.101187

**Published:** 2025-09-29

**Authors:** Luca Tosti, Calum Mould, Imogen Gatehouse, Anthony C. Smith, Krystian Ubych, Valentina Miano, Peter W. Laird, Jack Kennefick, Robert K. Neely

**Affiliations:** 1 Tagomics, Ltd., Cori Building, Granta Park, Cambridge CB21 6GP, UK; 2 Van Andel Institute, 333 Bostwick Ave. NE, Grand Rapids, MI 49503, USA; 3 The University of Birmingham, School of Chemistry, Edgbaston, Birmingham B15 2TT, UK

**Keywords:** DNA methylation, methyltransferase, cfDNA, epigenome, unmethylome, unmethylated DNA, liquid biopsy, tissue of origin

## Abstract

We present “Active-Seq” (azide click tagging for *in vitro* epigenomic sequencing), a base-conversion-free technology that enables the isolation of DNA containing unmodified CpG sites using a mutated bacterial methyltransferase enzyme and a synthetically prepared cofactor analog. Active-Seq is a robust epigenomic profiling platform with a simple and streamlined workflow, performed in tandem with sequencing library preparation and compatible with DNA input quantities as low as 1 ng. We establish a baseline for the performance of Active-Seq using model DNA oligos and further validate it against gold-standard whole-genome bisulfite sequencing data. We show robust performance of the platform across tissue-derived DNA and demonstrate enrichment of DNA at unmethylated, cell-type-specific marker regions of the epigenome, laying the foundation for the future application of this technology in tissue deconvolution applications. Finally, we apply the technology to cell-free DNA samples, outlining an approach for tumor-informed disease profiling in patients with colorectal cancer.

## Highlights

• Efficient, genome-wide profiling of unmodified DNA with as little as 1 ng input DNA
• Unique targeting and enrichment of unmethylated enhancers that define cell type
• Genome-wide, epigenomic profiling from as few as 70 million sequencing reads

## Motivation

Existing technologies for profiling DNA modifications focus on the detection of modified cytosine, which is typically associated with the inactivation of gene expression through promoter hypermethylation. However, DNA hypomethylation is a facet of early disease development, and unmethylated enhancers play an important role in defining cell-type identity. In this work, we sought to develop a robust method for genome-wide profiling of unmodified CpG sites.

## Graphical abstract

**Figure:**
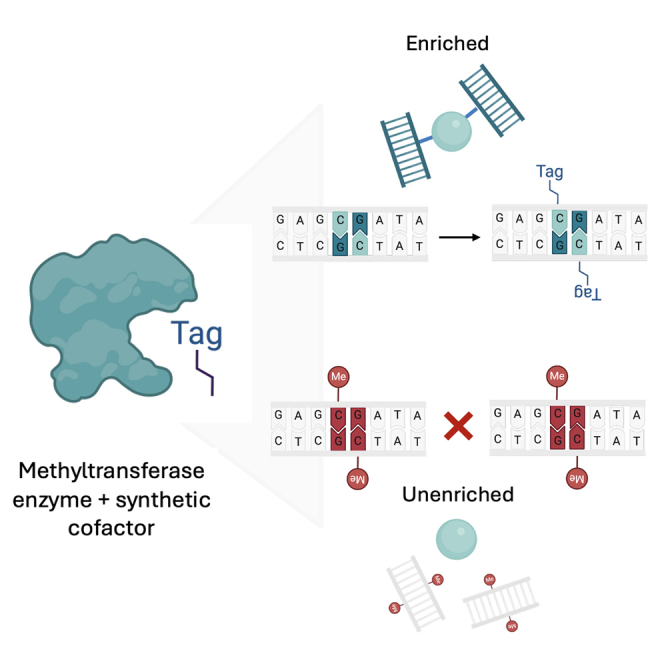

## Teaser

Tosti et al. report a method for genome-wide, epigenomic profiling that enriches DNA containing unmodified CpG sites for analysis. The enzymatic approach does not damage DNA, nor does it rely on base conversion, and it allows profiling of cell-free DNA with as little as 1 ng of input material.

## Introduction

Epigenetic modifications of DNA, such as methylation and hydroxymethylation of cytosine, play a pivotal role in gene regulation.^1^ Indeed, methylation of DNA is critical in embryogenesis and early development, and it is known to change predictably in correlation with the biological aging of an organism.^2^^,^^3^^,^^4^ On the other hand, aberrant methylation of DNA is an important driver of tumorigenesis, as the epigenetic alterations of gene regulatory regions play a key role in many diseases.^5^^,^^6^^,^^7^^,^^8^

Recent work by Loyfer et al. has shown the value of a genome-wide approach to epigenomic analysis, using whole-genome bisulfite sequencing (WGBS).^9^ Applied to seventy-seven primary cell types in healthy human cells, the obtained profiles show striking differences in the methylation patterns that are unique to a given cell type and unmethylated in the vast majority of cases. These cell-type-specific blocks of unmethylated DNA are strongly correlated with markers of active chromatin (active enhancers, H3K27ac, and H3K4me1) and, because of their uniqueness, allow the deconvolution of signals from multiple cell types in systems containing mixtures of cells (e.g., blood). Unmethylated markers for cell type represent a significant opportunity for identifying the tissue of origin for DNA in blood, but existing technologies, focused on the detection of methylation, are poorly suited to targeting unmethylated DNA.

Extended global hypomethylation of the genome was the first epigenetic process to be linked with cancer development.^10^^,^^11^ Since then, it has been shown that this process is driven in regions of the genome that replicate late in the cell cycle, where the native maintenance methylases are unable to keep pace with the rapid rate of cell division in tumors.^12^^,^^13^^,^^14^^,^^15^^,^^16^ This feature of the cancer genome has been linked to the early developmental stages of cancer through studies of methylation at LINE-1 elements (as well as other repetitive genomic elements), which act as a proxy for the genome-wide methylation level.^17^^,^^18^^,^^19^^,^^20^^,^^21^ The emerging picture reveals that DNA hypomethylation, or the loss of DNA methylation, is an activating marker that could be a critical driver and/or indicator of cell biology and early cancer development. However, this is again at odds with the current techniques for profiling DNA methylation, which ubiquitously focus on the detection of methylated bases.

Genome wide, comparative studies of the human DNA methylome have been largely carried out using array-based platforms, with the latest array covering 935,000 sites, i.e., around 3% of the 28 M CpG sites of the human genome.^22^ The focus on a small fraction of the DNA methylome limits the discovery of new methylation-based biomarkers and the study of large-scale regions of contiguous (un)methylation. Moreover, genome-wide methylation profiling technologies such as bisulfite conversion, enzymatic methyl sequencing (EM-seq)^23^ or Tet-assisted pyridine borane sequencing (TAPS)^24^ can be prohibitively expensive to implement at the population scale and suffer from significant sequencing redundancy (the whole genome is sequenced, yet there are only 56 M bases in the methylome). Enrichment-based approaches aim to address this, offering genome-wide analysis on a genomic fraction targeted for methylation marks (methylated DNA immunoprecipitation sequencing [MeDIP-seq] and methylated DNA binding domain sequencing [MBD-seq]). However, these non-covalent approaches show significant bias toward heavily methylated regions of the genome.^25^ Efforts have been made to reduce input amounts for these technologies,^26^^,^^27^ yet the fundamental nature of the preferential binding for densely methylated regions cannot be circumvented. Direct readout of methylation status during sequencing using nanopore sequencing offers promise, but for applications using cell-free DNA (cfDNA), the limited DNA available makes this extremely challenging.^28^

The discovery of novel DNA methylation-based biomarkers by Loyfer et al.^9^ after over 25 years of research effort in the field highlights the need to increase the spectrum of technologies that can be applied to facilitate the study of DNA methylation and fully resolve its complexity.

We have previously shown that the M.MpeI methyltransferase can be repurposed as a DNA alkyltransferase,^29^^,^^30^ and Gabrieli et al. have led the way on its use as a tool for the detection of unmodified CpG sites using a novel fluorescence-based assay.^31^ Here, we integrate this DNA alkylation step into a workflow for the enrichment and sequencing of unmodified CpG sites. The azide click tagging for *in vitro* epigenomic sequencing (Active-Seq) platform uniquely focuses on the analysis of unmethylated CpG sites (typically 20%–30% of the genomic CpG sites), allowing a scalable approach to genome-wide epigenomic profiling. Active-Seq makes a step change over pioneering work from Kriukienė et al.,^32^ which first demonstrated the enzymatic enrichment of unmethylated CpGs, with an improved workflow efficiency that enables, for the first time, the use of DNA inputs at the nanogram level. We demonstrate that this is transformative for the application of Active-Seq to the profiling of unmethylated DNA from liquid biopsy samples. The inherent simplicity of the approach means that Active-Seq is robust across laboratory operators and sequencing runs and can be used to generate genome-wide epigenetic profiles, with no assumed knowledge of the sample. Crucially, the enzyme-catalyzed chemistry preserves the genomic mutational signature, as well as fragmentomics information, i.e., features such as fragment size, end motif sequences, etc.,^33^^,^^34^ and, hence, has the potential to serve as the foundation for a streamlined, multiomic, and clinically deployable diagnostic tool.

## Results

### An integrated workflow for epigenomic sequencing

**Figure 1:**
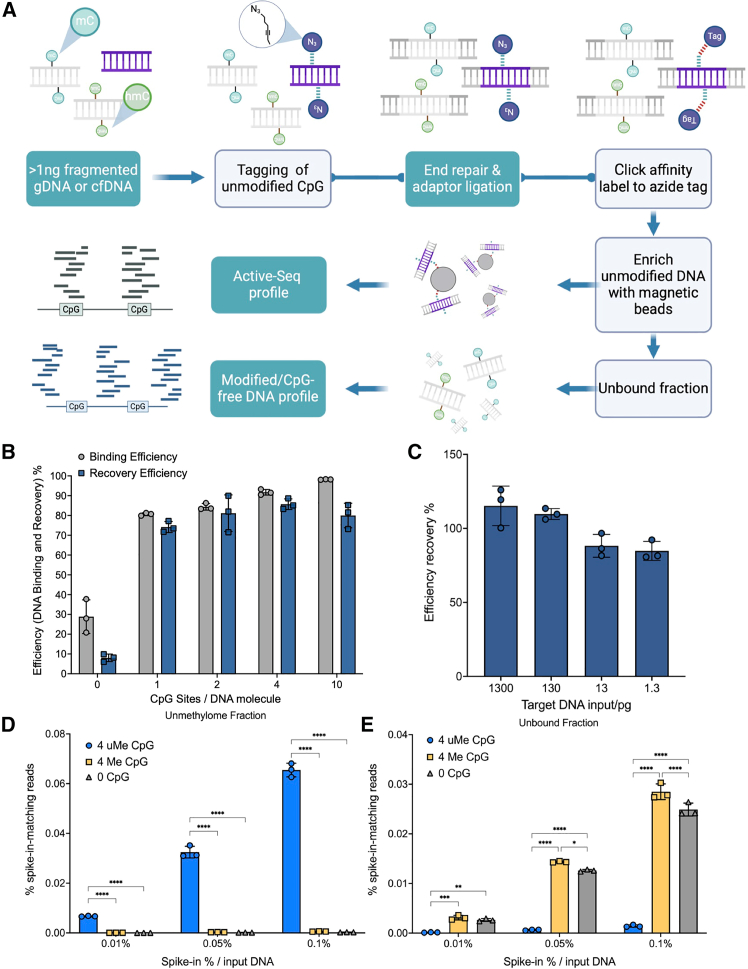
Schematic of the Active-Seq workflow and initial method validation using synthetic oligonucleotides (A) Schematic showing the single-tube, Active-Seq workflow. Purified DNA (cfDNA or fragmented gDNA) is tagged using a CpG-targeting methyltransferase (M.MpeI) and a synthetic cofactor analog. The enzyme catalyzes DNA alkylation with azide-terminated tags exclusively at unmodified CpG sites. Tagged DNA molecules are subsequently subject to standard library end repair and adapter ligation, followed by affinity tagging and isolation using streptavidin-coated magnetic beads. Tagged (unmodified CpG sites, Active-Seq) and untagged (5mCpG and other modified CpG sites and CpG-free fragments, unbound fraction) DNA can be separately amplified and sequenced. (B) Plot showing the efficiency for DNA binding and release (recovery) of tagged and affinity-labeled DNA, using 10 ng target DNA input, as a function of target DNA CpG site density. Binding efficiency is calculated based on the assumption that any reduction in DNA concentration after DNA incubation with beads is as a result of DNA binding to the beads. (C) Plot showing the efficiency for binding and release (recovery) of tagged and affinity-labeled DNA for a decreasing amount of input target DNA against a background of 24 ng of non-target DNA (containing no CpG sites). Note that the recovered DNA concentration was determined by qPCR, and reported recoveries are measured against DNA we expect to be bound to the beads (input DNA quantified via Qubit). (D) Plot showing number of reads of spiked-in DNA oligos (5, 25, and 50 pg in total) in the enriched (unmethylated) fraction with four unmethylated CpG sites (4 uMe CpG), four methylated CpG sites (4 Me CpG), and no target CpG sites (0 CpG) from a typical Active-Seq experiment. (E) Plot showing number of reads of spiked-in DNA oligos (5, 25, and 50 pg in total) in the unbound fraction with four unmethylated CpG sites (4 uMe CpG), four methylated CpG sites (4 Me CpG), and no target CpG sites (0 CpG) from a typical Active-Seq experiment. Data in bar-plots are presented as mean ± SD of three independent replicates. Two-way ANOVA test: ∗*p* < 0.05, ∗∗*p* < 0.01 , ∗∗∗*p* < 0.001 and ∗∗∗∗*p* < 0.0001.

The Active-Seq approach is described schematically in Figure 1A. We employ a bacterial DNA methyltransferase enzyme (M.MpeI) to tag unmodified CpG sites,^30^ enabling the downstream isolation of this fraction of the DNA. The DNA transalkylation reaction is achieved by substituting the enzyme’s native cofactor, *S*-adenosyl-*l*-methionine (AdoMet), with a synthetic cofactor analog, which enables DNA transalkylation with an azide-terminated functional group.^35^ Incubation of the methylase, DNA, and cofactor for 1 h results in near-complete modification of the target DNA (Figures S1 and S2). These tags can be further modified using highly efficient, bio-orthogonal click chemistry and an affinity tag^35^ to enable their capture (Figure 1A). Note that “unmodified” refers to all genomic DNA fragments containing at least one CpG dinucleotide that is not modified (methylated, hydroxymethylated, carboxylated, or formylated) at the C5 position. Active-Seq is a single-tube approach for tagging, enrichment, and DNA library preparation, designed with the overarching aim of minimizing handling and purification steps to maximize overall efficiency (transfer of DNA) through the workflow.

### Characterization of enzymatic DNA enrichment efficiency

In principle, DNA enrichment efficiency for a pool of DNA molecules is dependent on the number of available unmethylated CpG sites (a function of methylation status and CpG site density) and on the overall workflow efficiency (tagging, adapter ligation, affinity labeling, and enrichment). To understand the latter, we first established the performance of our enrichment approach using DNA fragments derived by PCR from the bacteriophage genome lambda, with known CpG site density (Table S1). To demonstrate efficient binding and release of DNA, we tagged DNA with a range of CpG densities (from 1 site/150 bp to 10 sites/150 bp), bound it to streptavidin-coated magnetic beads, washed and released it, and quantified this released DNA by fluorometric quantification (Figure 1B). The initial step of binding tagged DNA to magnetic beads shows a modest dependence on the number of CpG sites available for tagging, at low CpG site concentrations of less than 4/150 bp. We also note some non-specific binding of non-target DNA to the magnetic beads used for enrichment, though less than 5% of this material is eluted. Recovery efficiencies show no detectable dependence on the CpG site density, and overall recovery of the DNA is between 60% and 80% across the range of tested CpG densities.

To understand the specificity of this approach for the enrichment of small quantities of target DNA against a background of non-target DNA, we spiked between 1.3 ng and 1.3 pg of target DNA molecules (153 bp, containing 10 unmodified CpG sites) into a background of 24 ng of non-target DNA (142 bp containing no CpG sites). We tagged this mixed DNA using our enzymatic labeling and isolated the tagged DNA fraction using streptavidin-coated beads. The quantity of target DNA isolated was determined by qPCR, using primers specific to the target DNA molecules (Figure 1C; Table S1). We found DNA binding and recovery efficiencies of 80%–100% for all the samples, consistent with findings from our experiments (Figure 1B) using an input of pure target DNA. This clearly demonstrates the compatibility of the approach for enrichment of DNA at input levels consistent with cell-free DNA analysis.

To conclude this series of initial validation experiments, we examined the fate of target and non-target DNA oligos, spiked into a background of 50 ng of genomic DNA sample, as we processed it using the complete Active-Seq protocol (including library preparation). Following library preparation and enrichment, the enriched fraction of the sample contains on the order of 100-fold more of the unmethylated DNA spike-in than either of the negative control (methylated and non-target DNA) spike-ins (Figure 1D; Table S2). The number of reads from unmethylated spike-ins in this fraction decreases linearly with the spike-in quantity against the constant background of 50 ng of genomic material, demonstrating the recovery of the spike-in at 0.003% of the total sample input DNA. As anticipated, this indicates that the signal in the sequencing experiment is likely linearly dependent on the concentration of unmethylated DNA in the input sample. Furthermore, the fraction of false positive reads from the completely methylated and non-target spike-in oligos is less than 1% (∼0.6%) across the spike-ins tested (Table S2). Hence, with this complete, optimized workflow, we observe a significant improvement in the signal-to-noise ratio compared to the binding/recovery experiment (Figure 1B).

The unbound fraction contains the vast majority of the methylated and non-target DNA oligos, with the unmethylated spike-in almost completely depleted from this fraction of the sample (Figure 1E). In all, the Active-Seq enrichment is highly specific, regardless of the DNA input amount, and is, in principle, compatible with epigenomic profiling of cfDNA down to input levels (sub-ng) where library complexity begins to impact the fidelity of the epigenomic profile.

### Active-Seq performance in comparison to bisulfite sequencing

Active-Seq targets the 20%–30% of the CpG sites in the genome that are unmethylated, leading to a dramatic reduction of the required sequencing depth for genome-wide epigenomic profiling, compared to genome-wide approaches using base conversion. Genomic DNA isolated from the NA12878 cell line was fragmented, and DNA libraries were generated for the enriched (unmodified) and unbound (modified) fractions of the genome using Active-Seq.

The Active-Seq signal for unmodified DNA reaches 90% saturation at approximately 70 M reads (15 Gbp of sequencing per sample using 150 bp paired-end reads; Figure S3). Processed reads in Active-Seq show the levels of read duplicates (∼10%–15%) significantly below the levels observed in bisulfite sequencing (∼30%–40%) and similar to EM-seq (∼10%).^23^ Indeed, Active-Seq facilitates genome-wide epigenetic profiling with approximately 1/10^th^ of the sequencing depth recommended for bisulfite-converted samples (Table S3).^36^

**Figure 2:**
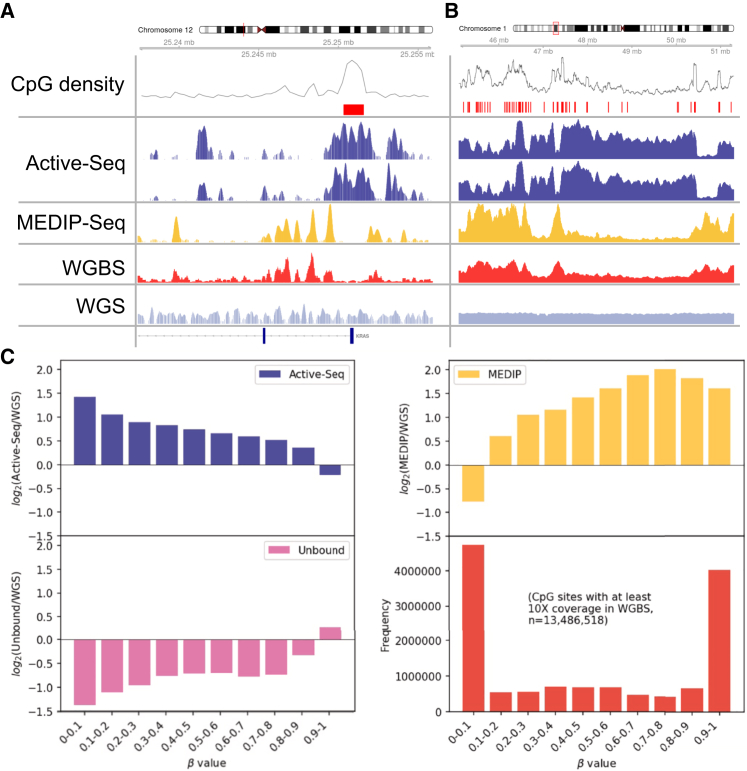
Active-Seq workflow and comparison with other epigenomics technologies (A) Active-Seq profiles (dark blue) in duplicate at the KRAS promoter, as compared with MeDIP-seq (yellow), whole-genome bisulfite sequencing (WGBS) signal (red), and whole-genome sequencing (WGS; gray) profile. (B) As in (A) but showing a larger (∼5 Mbp) region of the genome. (C) Log_2_ ratio of specific coverage vs. WGS coverage as a function of CpG site β-value, as determined by WGBS for Active-Seq (blue), the Active-Seq unbound (methylated) fraction (pink), and MeDIP-seq (yellow) in NA12878 cell line DNA. The red bar chart shows the number of CpG sites that lie in each of the β-value bins for a WGBS experiment (13.49 M sites in total, where CpG sites have been selected for only those with at least 10× coverage in the WGBS experiment). Active-Seq reads are binned in 100 bp windows and normalized to the sequencing depth (reads per genomic content [RPGC]).

We made a direct comparison of the Active-Seq signal to that defined by WGBS at isolated, unmethylated CpG sites. We used WGBS data^23^ to identify those isolated, unmethylated CpG sites with a β-value of zero and no other CpG site present within 250 bp up-/downstream. This produces a list of 25,852 CpG sites genome wide (Figure S4; full list of regions in Data S1). At these sites, the derived Active-Seq signal can be used to obtain an estimate of the Active-Seq enrichment efficiency by comparing it to the Active-Seq signal in the background regions of the genome containing no CpG sites (defined as a region of at least 1,000 bp in length that contains no CpG sites) (full list of regions in Data S1). We calculate an enrichment in Active-Seq, relative to background, for a DNA molecule containing a single unmethylated site to be approximately 160-fold (Figures 2A and 2B). Therefore, at sites defined as unmethylated by WGBS, Active-Seq shows high enrichment efficiencies, even where these sites are single, isolated CpGs.

The high enrichment efficiency of unmethylated sites in Active-Seq is clearly observed upon visual inspection of the Active-Seq profile in the genome browser (Figures 2A and 2B). The Active-Seq signal is anticorrelated to that of the methylation levels measured by WGBS and MeDIP-seq (Figures 2A and 2B) and is a true reporter of the genomic (un)methylation status.

To support the observations described above, we made a quantitative comparison of the Active-Seq signal to the genomic methylation level, as defined by the WGBS β-value, across 13.4 M CpG sites that had sufficient coverage (>10×) in the WGBS experiment. We measured the Active-Seq signal (normalized read count) as a function of the β-value and compared this to the similar metric for both the unbound fraction from the Active-Seq workflow and a MeDIP-seq experiment (yellow plots). Figure 2C shows these values plotted as a ratio, relative to the normalized read counts derived using whole-genome sequencing. As anticipated, the Active-Seq signal falls gradually with increasing methylation levels until it drops below the signal of the WGS experiment at highly methylated CpG sites (with β-values greater than 0.9). The magnitude of the normalized Active-Seq signal is in line with that observed for MeDIP-seq. Furthermore, the ratio of Active-Seq signal to WGS signal increases consistently as the methylation level falls (Figure 2C, blue and yellow), where the ratio of the MeDIP-seq signal to the WGS signal seems to peak at a β-value of 0.7–0.8 and fall at higher β-values (Figure 2C, yellow), suggesting some bias toward enrichment of sites with a β-value between 0.7 and 0.8. The Active-Seq signal is pleasingly mirrored by that of the unbound fraction from the Active-Seq workflow (Figure 2C, blue and pink).

**Figure 3:**
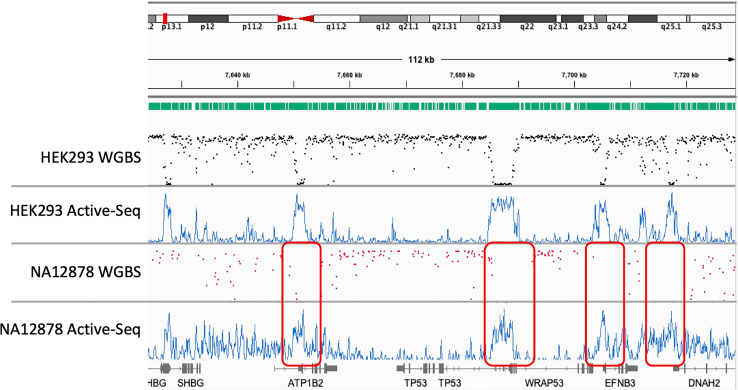
Genome browser screenshots showing comparison of Active-Seq data to data derived from whole-genome bisulfite sequencing at CpG sites Whole-genome bisulfite sequencing data (β-value) of DNA derived from the HEK293 cell line (black dots) (β-values determined using ∼750 M reads, only CpG sites with greater than 10-fold coverage are shown in the plot). Active-Seq unmethylome profile (blue traces) for DNA derived from the HEK293 cell line (∼470 M reads). Whole-genome bisulfite sequencing data (β-value) of DNA derived from the NA12878 cell line (red dots). Sequencing to ∼650 M reads with only CpG sites with greater than 8-fold coverage is displayed in the plot. Note that even with this sequencing depth, poor coverage of many CpG sites is limited (red boxes), preventing reliable β-value determination and downstream analysis. Active-Seq unmethylome profile (blue trace) for DNA derived from the NA12878 cell line (∼157 M reads). Green bars across the top of the figure show the location of individual CpG sites. Active-Seq reads are binned in 100 bp windows and normalized to the sequencing depth (reads per kilobase per million mapped reads [RKPM]).

Figure 3 shows example Active-Seq profiles, compared to analogous WGBS experiments for two cell lines, at different sequencing depths. The Active-Seq signal is continuous, in contrast to the punctate data derived from WGBS, highlighting the CpG site resolution of the latter, relative to Active-Seq, where the resolution of the profile is dictated by the size of the captured, unmodified DNA fragments. However, in Active-Seq, in the absence of base conversion, reads are efficiently mapped to the reference genome with no loss of genomic sequence complexity.^23^ The impact of this is that, despite high average genomic coverage (typically ∼30×), some regions of the genome have low read counts in WGBS, particularly those with high GC content that have low sequence complexity following base conversion. Typically, CpG sites with less than 10-fold coverage in the converted or unconverted sample will be removed from the final dataset (Figure 3), and this can result in patchy coverage of CpG sites across the genome (e.g., as indicated by the red boxes in Figure 3 for the dataset of the NA12878 cell line). Further example screenshots of these datasets can be seen in Figure S5.

The signal in an Active-Seq profile is dependent on three factors: (1) unmodified CpG density in a genomic region, (2) the DNA concentration in the sample at that genomic region, and (3) the overall workflow efficiency. We use saturating conditions for the enzymatic tagging step and thus expect workflow efficiency to be similar in all cases. However, the concentration of unmodified CpG sites (unmodified CpG density × DNA concentration) can vary significantly at a given genomic region, depending on the source of the DNA (formalin-fixed paraffin-embedded [FFPE], cfDNA, or tissue/cell line). In a tissue- or cell-line-derived DNA where the sample shows a uniform DNA concentration across the genome, we expect that the Active-Seq signal will be inversely correlated to the β-value derived using WGBS (Figure 3).

Sequencing coverage in Active-Seq was compared to that of WGBS and MeDIP-seq in regions of the genome that are enriched in histone marks for active chromatin (Figure S6A). These regions are associated with genomic enhancers (H3K4me1 and H3K27ac) and promoters (H3K4me3) and can be aberrantly methylated in disease.^34^ Active-Seq shows a strong signal in these regulatory regions, in contrast to the depletion of reads in the corresponding MeDIP-seq and WGBS experiments (Figure S6A).

To comprehensively characterize the distribution of the Active-Seq signal over functional genomic regions, we analyzed the signal profile across 15 chromatin states previously defined in the NA12878 cell line.^37^ Active-Seq shows enrichment at transcription start sites (TSSs; and flanking regions), enhancers, bivalent chromatin, and regions repressed by the Polycomb repressive complex (PRC) (Figure S6B). Furthermore, the Active-Seq signal is depleted in regions known to be rich in methylated DNA (intragenic regions of strongly and weakly transcribed genes) and in quiescent/lowly active areas of the genome. Note that, consistent with their highly methylated nature, the Active-Seq profile is also depleted in gene bodies that are transcribed (Figure S6B, “strong transcription” and “weak transcription” in the ChromHMM 15 state model).

### Active-Seq signal correlates with unmethylated cell-type-specific markers, identified using bisulfite sequencing

**Figure 4:**
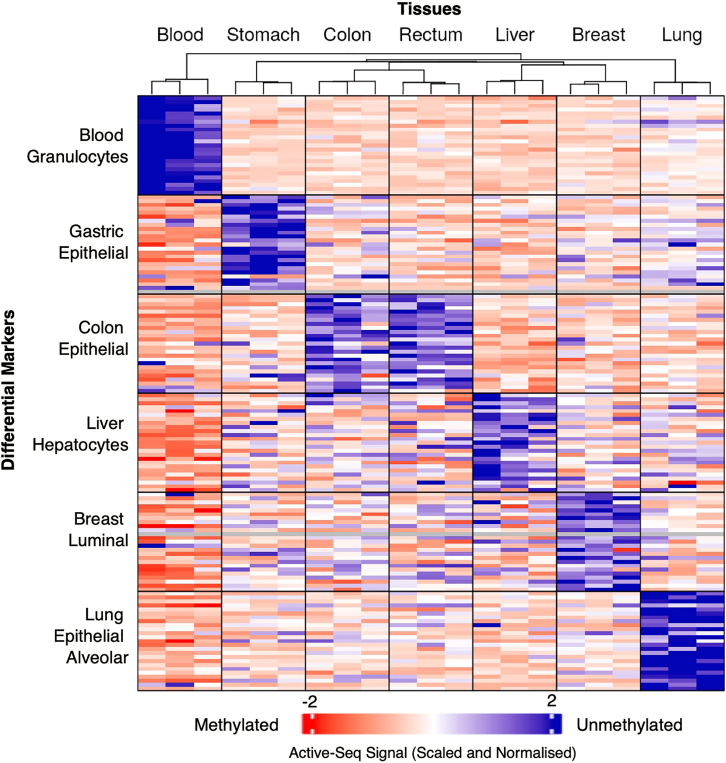
Active-Seq signal at tissue-specific marker regions Active-Seq enrichment at cell-specific differentially methylated regions (top 25 regions/tissue identified in Loyfer et al.^9^). Samples are three technical repeats of Active-Seq, except for the blood samples, which are derived from the Active-Seq profiles of three individual patients (buffy coat). Tissue-specific differentially methylated regions are grouped according to the tissue/cell type with which they are associated.

We evaluated the Active-Seq signal in normal adjacent tissue samples at tissue-specific unmethylated markers from the methylation atlas of healthy human cell types defined by Loyfer et al.^9^ These markers are methylation blocks of three or more adjacent unmethylated CpG sites and provide a unique epigenetic signature for a given cell type (being specifically unmethylated in one cell type vs. all other cell types). Figure 4 shows the enrichment of the Active-Seq signal in the unmethylated marker regions defined by Loyfer et al.^9^ for our six normal adjacent tissues. We successfully detected tissue-specific signals despite the heterogeneity of the bulk tissue samples used in our experiments, compared to the highly purified, sorted cell types used by Loyfer et al.^9^ The robustness of these markers, combined with the ability of Active-Seq to enrich for unmethylated regions of the genome, enabled us to detect a strong, tissue-specific epigenetic signature in these healthy tissues. These results pave the way for future applications of Active-Seq aimed at identifying the tissue of origin of a given sample.

### Active-Seq targets cell-type- and tissue-specific epigenomic markers

**Figure 5:**
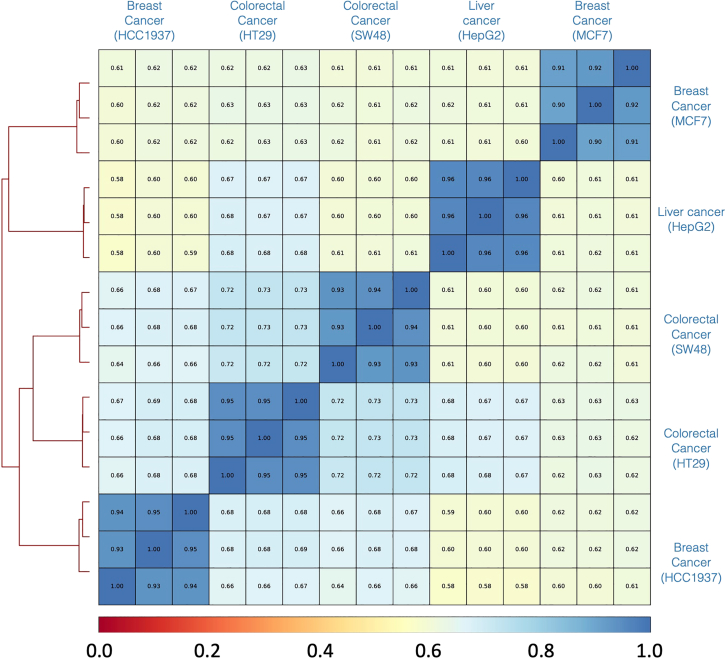
Genome-wide analysis of the Active-Seq profile for five different cell lines in 1,000 bp windows across the genome Plot showing results of unsupervised clustering of Active-Seq profiles (left). The matrix shows the Spearman correlation for each of the of Active-Seq profiles, in three technical repeats, generated from five different cell lines.

Having characterized the genomic features of the Active-Seq signal, we explored its ability to detect differences in profiles from biologically distinct samples. We applied Active-Seq to five cell lines derived from a range of cancers, namely breast (MCF7 and HCC1937), colorectal (HT29 and SW48), and liver (HepG2). A genome-wide Spearman correlation analysis of this dataset shows excellent correlation (0.91–0.96) of each series of three technical repeats for each of the samples, confirming the robustness of the Active-Seq workflow (Figure 5). Each of the cell lines forms a distinct cluster, with cell types from related tissues (colorectal cancer [CRC]) clustering together. Furthermore, while the HCC1937 and MCF7 cell lines are both derived from breast tissue, they were isolated from quite distinct regions of the tissue: basal/mesenchymal and luminal epithelial, respectively.^38^ This suggests the Active-Seq profile is sensitive to the cell type, in line with the reported observation that unmethylated enhancers define cell type.^9^

We found similarly consistent clustering of the Active-Seq signal for three pairs of tumor and normal adjacent tissue samples (Figure S7). Unsupervised clustering of these data shows distinct clustering of the cancer and normal adjacent tissue profiles, with the technical repeats for each sample clustering together. This simple analysis makes no prior assumptions about the dataset and demonstrates the potentially discriminative ability of Active-Seq for both tissue type and disease status from patient samples.

### Active-Seq targets genomic regions that are clinically informative

**Figure 6:**
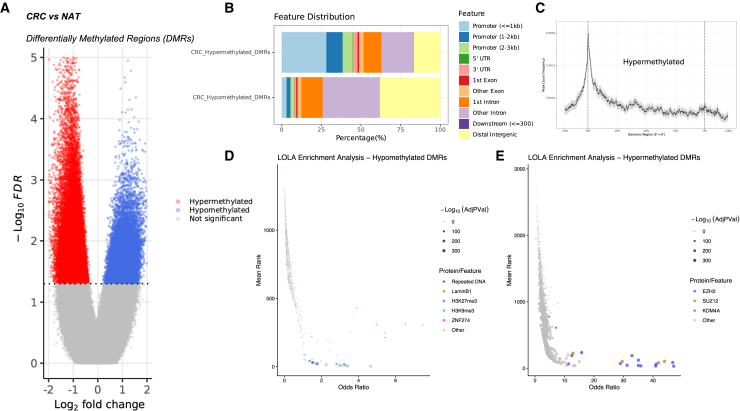
Differentially methylated regions identified in solid tumor samples from seven patients with colorectal cancer (A) Volcano plot of hypo- (blue) and hypermethylated (red) differentially methylated regions (DMRs). (B) Genomic feature annotation of hypo- and hypermethylated DMRs. (C) Frequency profile of hypermethylated DMRs across gene features. (D) LOLA enrichment analysis^39^ of hypermethylated DMRS. (E) LOLA enrichment analysis of hypomethylated DMRs.

We performed a series of experiments using tumor and corresponding normal adjacent tissue from seven patients with CRC as a proof of concept of the potential of Active-Seq as a diagnostic tool. The comparison of Active-Seq profiles from tumor and normal adjacent tissue (see the STAR Methods for full details) requires no prior knowledge of a patient’s genomic sequence and makes no assumptions about regions of interest in the genome. From this comparison, we identified 47,420 significantly hypermethylated regions and 28,080 significantly hypomethylated regions in the tumor tissue (Figure 6A). As anticipated, these hypo- and hypermethylated differentially methylated regions (DMRs) cover distinct genomic features. Hypermethylated DMRs overlap significantly with TSSs, while hypomethylated DMRs cover mainly intergenic regions and introns (Figures 6B and 6C).

We then performed a locus overlap analysis (LOLA)^39^ of the hypo- and hypermethylated DMRs with genomic features and transcription factor binding sites (TFBSs) associated with this region set. The CRC hypermethylated regions showed a significant overlap with TFBS of PRC2, namely EZH2 and SUZ12, and of KDM4A, an H3K9me3 demethylase (Figure 6D). PRC2 is the key enzymatic complex responsible for the methylation of H3K27, a modification associated with gene repression. Targets of PRC2 are predisposed to hypermethylation in adult cancer cells, as a result of these H3K27me3 marks.^40^^,^^41^^,^^42^

The Active-Seq hypomethylated DMRs show significant overlap with repetitive DNA regions and H3K9me3-enriched regions, in agreement with the ability of Active-Seq to target partially methylated domains (PMDs) (Figure 6E).^15^^,^^16^^,^^43^ Collectively, our results demonstrate that unbiased and genome-wide profiling of DNA using Active-Seq allows assessment of both the hypermethylation of promoters—traditionally used to inform patient stratification and prognosis in CRC^44^^,^^45^^,^^46^—and the genome-wide hypomethylation, which has been widely used as an early indicator of the disease.^18^^,^^47^^,^^48^^,^^49^^,^^50^

### Tumor-informed Active-Seq profiling in liquid biopsy samples

**Figure 7:**
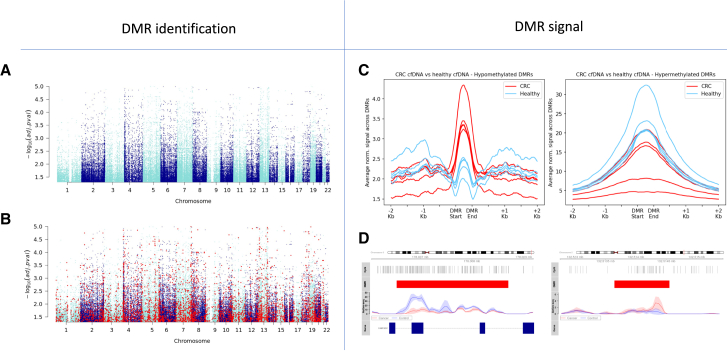
Active-Seq signal in liquid biopsy samples (A) Manhattan plot showing approximately 75,000 differentially methylated regions (DMRs) identified in colorectal patient samples (cohort of 7 patients, tumor and normal adjacent tissue). (B) DMRs in blue identified in (A) shown with ∼4,300 overlapping DMRs identified in the blood (circulating cell-free DNA, red dots) of five of these patients. (C) Average signal in blood samples at hypo- (left) and hypermethylated (right) DMRs for patients with colorectal cancer (red) and patients without colorectal cancer (blue). (D) Example profiles of individual DMRs in blood samples (red blocks) for patients with colorectal cancer (red solid lines) and healthy patients (blue) for hyper- (left) and hypomethylated (right) DMRs. Standard deviation for the signal in each cohort is given by the shaded bands.

Active-Seq can be applied using an identical workflow on DNA derived from both solid tumor and liquid biopsy samples. As such, it is ideally positioned as a tool for personalized tumor profiling (solid tumor) and subsequent monitoring of disease recurrence or treatment outcome using tumor-informed profiling of liquid biopsy samples. To test this hypothesis, we evaluated the Active-Seq signal in the 75,000 DMRs defined in our analysis of CRC tissues (Figure 7A) and found that for a subset of these, an average difference in signal (cancer vs. healthy) of greater than (±)1.5-fold was observed in cfDNA/blood samples (approximately 4,300 out of 75,000 DMRs; Figure 7B). Using this list of DMRs, we evaluated the average signal lying in these regions in Active-Seq profiles for cell-free DNA derived from plasma samples of a cohort of ten patients (five patients with CRC and five risk-matched healthy patients) (Figure 7C). Input DNA amounts for the cfDNA samples ranged from 1.5 to 10 ng (Figure S8).

The average signal generated across the hypomethylated DMRs clearly separates the healthy and CRC patient cohorts (Figure 7C, left). At hypermethylated DMRs, separation of the CRC and healthy cohorts is less clear, potentially indicating more variability in hypermethylated regions for individual patients (Figure 7C, right). Examples of individual DMR signals in the patient profiles derived from cfDNA are shown in Figure 7D. While the patient cohort is small in this case, the observation of a signal in cfDNA, using a list of DMRs identified in tissue (tumor vs. normal adjacent tissue), is consistent with previous work in early detection^5^ and gives confidence that the Active-Seq profile retains this biologically critical information.

## Discussion

Active-Seq is an enrichment-based epigenetic profiling technology that, uniquely, focuses on unmethylated CpGs. Of approximately 28 M CpG sites in the genome, an estimated 20%–30% of these are unmodified; hence, by enriching this fraction of the genome for analysis, we are able to generate a genome-wide, epigenomic profile with a fraction (around 1/10^th^) of the sequencing needed for a comparable base-converting approach. The regions enriched in Active-Seq are typically regulatory regions of the genome and particularly unmethylated regulatory regions that typically indicate an active transcriptional state for their associated genes. Furthermore, a subset of these regions, the unmethylated enhancers, are associated with a specific cell type and represent an epigenomic fingerprint that defines cell identity. Indeed, recent work has shown that these regions of the genome represent over 97% of the cell-type-specific methylation markers of the healthy epigenome.^9^ Variations in cell composition can obscure or confound the detection of true disease-related epigenetic changes,^51^ particularly in complex mixtures such as plasma/cfDNA. Hence, the unique targeting of these cell- or tissue-specific “signature” regions with Active-Seq will facilitate efforts to improve the quality of epigenomic biomarkers in the future.

Markers for active chromatin also correlate with the regions of unmethylated DNA enriched by Active-Seq. Enhancers are partially (10%–60%) methylated in healthy cells, and their differential methylation is, in fact, the prevalent perturbation to methylation of DNA at “functional” genomic elements.^52^^,^^53^^,^^54^ In cancer, hypomethylation of enhancers is more common, being approximately twice as prevalent, in a broad range of cancers, as enhancer hypermethylation.^52^ Furthermore, the accumulation of enhancer hypomethylation has been shown to correlate with the likelihood of metastasis and poor prognosis in lung,^55^ breast,^56^ and liver cancer, where hypomethylation of enhancers targeted by CCAAT/enhancer-binding protein-beta correlates with poor outcomes.^57^ This is supported by recent single-molecule work that has shown a functional link between DNA methylation status at a subset of enhancers and the efficiency of transcription factor binding at these sites.^58^

The phenomenon of DNA hypomethylation has long been considered a characteristic feature of the cancer genome.^17^^,^^18^^,^^19^^,^^20^^,^^21^ There is an established link between global and intragenic hypomethylation of the genome, typically measured at LINE-1 elements, which has been associated with poor prognosis in patients with gastric cancers and patients with advanced colorectal adenomas^47^^,^^48^^,^^59^ and has been used to monitor the efficacy of DNMT1 inhibitors in clinical trials.^60^ Recent works have localized this long-range hypomethylation of DNA to regions known as PMDs, which can be up to megabase-scale domains that correlate with the transcriptionally repressed genes that predominantly lie within the B compartment of the genome.^61^ Hypomethylation of the PMDs is thought to occur because of their late replication in the cell cycle and is exaggerated in rapidly dividing, cancerous cell populations, as highlighted in a study of over 1,500 patients with breast cancer.^13^ However, as a result of their low CpG density, these sites are poorly enriched in MeDIP-seq and MBD-seq, and variation in their methylation level, which tends to be of the order of 10%–20% across a broad genomic region, is challenging to measure using base-conversion approaches. Active-Seq instead enables access to these diagnostically useful regions of the genome, which provide an underutilized methylation signature of cancer.

The hypermethylation of specific gene promoters in cancer has been far more extensively studied than the process of genome-wide hypomethylation, which is also a hallmark of cancer.^62^ Promoters (linked to H3K4me3 histone mark modification) are stably unmethylated in a healthy cell and are highly enriched in the Active-Seq profile. The abnormal methylation of promoters provides a clear and distinct signature of disease, where methylation is typically correlated with gene inactivation. Using Active-Seq, we have identified thousands of significantly hypermethylated regions in tumor-derived tissue in the CRC study we performed.

Active-Seq provides a unique tool for studying unmodified regions of the genome. The information-rich profile is assumption free and genome wide and targets active regulatory elements and epigenomic regions that are suited to cell-type deconvolution. The workflow is robust and straightforward to apply in the laboratory. The enzymatic approach does not damage DNA, nor does it rely on base conversion. A complete Active-Seq epigenomic profile requires as little as 15 Gbp of sequencing per sample, with no requirement for up-front assumptions about the genomic regions of interest, as is implicit in the use of a panel for genome-wide profiling at the population level using base-conversion technologies. We have developed the Active-Seq enzymatic chemistry to enable unbiased enrichment of unmethylated DNA at DNA concentrations that, for the first time, are compatible with the profiling of cell-free DNA.

Active-Seq has a unique ability to enrich unmethylated DNA across both the short, highly unmethylated promoter and enhancer regions that are so critically linked to cancer progression and prognosis and the broad, partially methylated regions of the genome that likely become hypomethylated due to the rapid rates of cell division in tumors. As a result, the technology provides an information-rich, genome-wide profile that we anticipate will enable improved detection and characterization of disease, at scale.

### Limitations of the study

As with each technology platform for the study of DNA methylation, Active-Seq currently has limitations and opportunities for future improvements to be introduced. For example, currently, the resolution of the epigenomic profile is limited to the size of the captured DNA, since for DNA molecules with multiple CpG sites, we cannot be certain which site was unmethylated and led to the capture of a given molecule. However, since the capture of DNA is probabilistic and directly dependent on the DNA methylation status, it will be possible to deconvolve the profile structure at a given location to provide an assessment of methylation levels at individual CpG sites in the future. Related to this, and unlike base-converting technologies, Active-Seq does not provide a quantitative assessment of methylation status in the sample. Measurements are comparative, and the analysis focused on the direction and magnitude of changes in methylation status between conditions.

Our initial validation of the Active-Seq workflow in patient plasma samples demonstrates the compatibility of the workflow with liquid biopsy samples and that the derivation of a genome-wide epigenomic profile for patients with low levels of cfDNA available (down to 1 ng) is feasible. In the future, we will further extend this study to a broader patient cohort with a focus on leveraging the genome-wide profiling from Active-Seq to enable the early detection of disease.

## Resource availability

### Lead contact

Requests for further information and resources should be directed to and will be fulfilled by the lead contact, Dr. Robert K. Neely (r.k.neely@bham.ac.uk).

### Materials availability

The Tagomics Activace Kit for performing Active-Seq will be made available through an early access program and may require a payment and/or a completed materials transfer agreement.

### Data and code availability

• Data used for the method validation (profiles derived from cell line DNA) have been deposited in GEO with the accession number GEO: GSE266366 and are publicly available as of the date of publication. Data derived from patient samples are available from the European Genome Archive (EGA) with study accession number EGAS50000001226.
• This paper does not report original code.
• Any additional information required to reanalyze the data reported in this paper is available from the lead contact upon request.

## Acknowledgments

The authors would like to thank Dr. Linda Sher and Dr. Sang Won Lee, Keck School of Medicine, USC, for their kind support and feedback on the manuscript. The Active-Seq method was developed using funding from the 10.13039/501100000266Engineering and Physical Sciences Research Council (EPSRC) grant number EP/N020901/1.

## Author contributions

C.M., I.G., K.U., and V.M. performed the experiments; L.T. and A.C.S. designed and implemented the bioinformatic analysis; C.M., I.G., V.M., K.U., J.K., and R.K.N. designed the experiments; L.T. and R.K.N. wrote the manuscript; and J.K., P.W.L., L.T., V.M., and R.K.N. reviewed and edited the manuscript and contributed to the study design.

## Declaration of interests

L.T., C.M., V.M., A.C.S., R.K.N., and J.K. are employees of Tagomics. J.K. and R.K.N. are directors for and hold shares in Tagomics. K.U., J.K., and R.K.N. have patent applications that have been licensed from the University of Birmingham to Tagomics. P.W.L. is a scientific advisor for Tagomics. Tagomics is developing the Active-Seq platform for commercial application in biomarker discovery.

## STAR★Methods

### Key resources table

**Table:** 

REAGENT or RESOURCE	SOURCE	IDENTIFIER
**Biological samples**		

Human colorectal cancer tissue sections	Keck School of Medicine Biorepository	https://keckbiobank.med.usc.edu
Human plasma from control/colorectal cancer patients	Keck School of Medicine Biorepository	https://keckbiobank.med.usc.edu
Human Matched Pair gDNA - Breast	Biochain	cat#: D8235086-PP-10
Human Matched Pair gDNA - Colon	Biochain	cat#: D8235090-PP-10
Human Matched Pair gDNA - Liver	Biochain	cat#: D8235149-PP-10
Human Matched Pair gDNA - Lung	Biochain	cat#: D8235152-PP-10
Human Matched Pair gDNA - Rectum	Biochain	cat#: D8235206-PP-10
Human Matched Pair gDNA - Stomach	Biochain	cat#: D8235248-PP-10

**Chemicals, peptides, and recombinant proteins**		

Tris Base	Sigma Aldrich	cat#: T1378
Tween 20	Fisher Bioreagents	cat#: BP337
Hydrochloric Acid	Sigma Aldrich	cat#: H1758

**Critical commercial assays**		

Activace Kit for Unmethylome Enrichment	Tagomics	https://tagomics.com/partners/
Qubit™ 1X dsDNA High Sensitivity (HS)	invitrogen	cat#: Q33231
Novaseq 6000 - S4 (300 Cycle) Sequencing Kit	Illumina	cat#: 20028312
Lambda phage DNA	NEB	cat#: N3011L
KAPA HyperPrep Library Prep	Roche	cat#: KK8504
8 microTube-50 AFA Fiber V2 Strips	Covaris	cat#: 520174
AMPure XP Beads	Beckman Coulter	cat#: A63882
Unique Dual Indexed Primer Pairs	Intergrated DNA Technologies (IDT)	cat#: 10005922

**Deposited data**		

Public WGBS Data for NA12878	https://doi.org/10.17989/ENCSR890UQO	GEO: GSE86765
Active-Seq data for human gDNA derived from cell lines	This paper	GEO: GSE266366
Active-Seq data derived from patient cell-free DNA	This paper	EGA: EGAS500000012266

**Experimental models: Cell lines**		

Human gDNA: HCC1937	ATCC	cat#: CRL-2336D
Human gDNA: HT29	ATCC	cat#: HTB-38D
Human gDNA: SW48	ATCC	cat#: CCL-231DQ
Human gDNA: HepG2	ATCC	cat#: HB-8065D
Human gDNA: MCF7	ATCC	cat#: HTB-22D
Human gDNA: NA12878	Coriell Institute	cat#: NA12878

**Oligonucleotides**		

Primers/Sequences for control PCR fragments, see Table S1	This paper	N/A

**Software and algorithms**		

Azure Cielo Manager Analysis Software (V1.0.4)	Azure Biosystems	https://azurebiosystems.com/products/cielo/
BBTools	Bushnell^63^	sourceforge.net/projects/bbmap/
BWA-MEM2	Vasimuddin et al.^64^	https://github.com/bwa-mem2/bwa-mem2
SamTools	Danecek et al.^65^	https://www.htslib.org
Sambamba	Tarasov et al.^66^	https://lomereiter.github.io/sambamba/
jvarkit	Lindenbaum	https://github.com/lindenb/jvarkit
deepTools	Ramirez et al.^67^	https://deeptools.readthedocs.io/en/latest/
QSEA	Lienhard et al.^68^	https://bioconductor.org/packages/release/bioc/html/qsea.html
REpitools	Statham et al.^69^	https://bioconductor.org/packages/release/bioc/html/Repitools.html
featureCounts	Liao et al.^70^	https://subread.sourceforge.net/featureCounts.html
DESeq2	Love et al.^71^	https://bioconductor.org/packages/release/bioc/html/DESeq2.html
DiffSegR	Liehrmann et al.^72^	https://aliehrmann.github.io/DiffSegR/index.html

### Experimental model and study participant details

#### Patient DNA samples

Samples from patients (colorectal cancer and non-cancer controls) were provided by Keck School of Medicine of the University of Southern California with approval of the USC Institutional Review Board. The small cohort of patient samples were selected for analysis from IRB approved repositories (HS-18-001049 and HS-028017) from participants in those repositories that have provided consent to allow samples to be shared and that have allowed their records to be accessed to obtain the data being collected for this study. The patient cohort consists of seven patients diagnosed with colorectal cancer, for whom tumor, normal adjacent tissue (both stored as fresh, frozen tissue) were profiled (three male and four female patients). Of these patients, five also had plasma available, from which cell-free DNA was extracted and profiled. Average age of patients was 62, with a spread from 53 to 74 years old. Plasma from five risk-matched patients without colorectal cancer were used as controls for the study. Average age of the control samples was 56 with a spread from 47 to 76 years old (four male and one female patient). Patient tumor staging is given in Data S1.

Genomic DNA from cell lines was sourced from ATCC and Coriell Institute (see Table above).

### Method details

#### Sample preparation

cfDNA extraction from clinical samples was performed by Informed Genomics, concentration of extracted cfDNA were determined using fluorimetry (Qubit, High Sensitivity) and quality was assessed using a TapeStation (High Sensitivity D1000 Tape). Genomic DNA was extracted from frozen tissue samples and FFPE samples by YourGene Health. DNA concentrations were determined using fluorimetry (Qubit, High Sensitivity). Following extraction, genomic DNA was sheared to an average of 180 bp in 8 microTube-50 AFA Fiber V2 Strips using a Covaris E220 evolution sonicator. 10 of cfDNA or 50 ng tissue-derived (stored in FFPE or fresh frozen) DNA was used as input for Active-Seq experiments.

Production of M.MpeI and its cofactor analogue were outsourced and produced according to the procedure described in Wilkinson et al.^30^ Quality control of both enzyme and cofactor analogue was performed by incubating MpeI and cofactor analogue with 100 ng pUC19 DNA in buffer (NEB Cutsmart) for 1 h, Figure S1. MpeI or cofactor analogue were subject to a serial dilution, to determine the minimum concentration of enzyme and cofactor required for complete protection of 100 ng DNA in one hour. We define this a ‘unit’ of reagent and perform all DNA modification reactions using 1 unit of methylase and cofactor.

DNA tagging, and enrichment was performed using the Tagomics Activace kit for unmethylome enrichment. Briefly, DNA was combined with 1 unit of M.MpeI methyltransferase enzyme, buffer (NEB Cutsmart) and 1 unit of methyltransferase cofactor analogue in a 20 μL reaction volume (Figure S2). This solution was incubated at 37°C for one hour. Following DNA tagging, end-repair and adapter ligation was performed (KAPA Hyper Prep kit), according to the manufacturer’s instructions. Tagomics affinity tag was added to the DNA and incubated at 37°C for an hour. Affinity tagged DNA was subsequently purified from the reaction mixture using AMPure beads (Beckman Coulter) and then bound to streptavidin-coated magnetic beads (ThermoFisher) for enrichment. Isolated, enriched DNA was transferred to a sterile tube and either stored at −20°C or used directly for amplification. A mixture of KAPA HiFi HotStart ReadyMix (2x, 25 μL), IDT Unique Dual Index Primer Pairs (5 μL of 10 μM Stock) and the enriched DNA library (20 μL) was prepared and subjected to amplification by PCR. The amplified library was purified using AMPure XP Beads and eluted in 10mM Tris-HCl (pH 8.5), 0.01% Tween 20. Libraries were pooled together with 1% PhiX and sequenced on an Illumina S4 flow cell on the NovaSeq platform (Source Biosciences).

#### Quantification of binding/recovery efficiency of tagged DNA

10ng of lambda PCR fragments (sequences available in Table S1) containing either 0, 1, 2, 4 or 10 CpGs within a 150bp molecule were prepared by PCR. The Tagomics Activace kit was used for unmethylome enrichment, according to the manufacturers instructions but with the omission of the library preparation (end-repair, A-tailing and adapter ligation) steps. Binding efficiency was calculated by quantifying (via HS dsDNA Qubit) the DNA concentrations before and after binding. The resulting change in DNA concentrations was assumed to be DNA binding to the Streptavidin Beads. Recovery efficiency was calculated through fluorometric quantification of DNA (ng) in release and presented as a percentage of DNA that went into binding.

#### qPCR

For each 20 μL qPCR reaction, 1 μL DNA sample, 400nM each of forward/reverse primers specific to the target fragment (see Table S1 for sequences) and 10 μL qPCR mastermix were prepared in triplicate. qPCR was performed on the Azure Cielo 6 thermocycler (Azure Biosystems) with the following conditions: initial denaturation at 98°C for 30 s, then 40 cycles at 95°C for 10 s and 60°C for 60 s with fluorescence detection. Analysis of the acquired fluorescence intensity and subsequent quantification of DNA in the samples was performed using Azure Cielo Manager Analysis Software (V1.0.4).

#### Processing and alignment of Active-Seq sequencing reads

After sequencing, adapters were removed from the reads using BBTools^63^ and then aligned to human reference genome HG38 using BWA-MEM2.^64^ Ambiguously aligned reads and those with low mapping scores (MAPQ score <40) were removed using SamTools.^65^ Duplicates were removed with Sambamba (Tarasov et al.^66^) and reads hard-clipped using jvarkit (https://github.com/lindenb/jvarkit). Spearman correlation plots were generated from the processed bam files with deepTools^67^ using a binsize of 1000bp and RPGC normalisation. Saturation figures and CpG density plots were generated for Chr1-22 using the QSEA^68^ and Repitools^69^ R packages. To allow direct comparison of enriched and unenriched samples, Bam files were down sampled to the same sequencing depth using SamTools. High confidence methylated and unmethylated CpG sites used for comparison were taken from the consensus of two whole genome shotgun bisulphite sequencing (WGBS) datasets performed on cell line NA12878 by the same lab (https://www.encodeproject.org/experiments/ENCSR890UQO/), where less than 5% methylation in both datasets was considered to be unmethylated and greater than 95% methylation in both datasets was considered to be methylated.

### Quantification and statistical analysis

#### Enrichment of spike-in DNA oligos over input DNA

Data in bar-plot in Figures 1D and 1E are presented as mean ± SD of three independent replicates. Significance was determined using a two-way ANOVA (Analysis of Variance) with Tukey’s multiple comparisons test. ∗*p* < 0.05, ∗∗*p* < 0.01, ∗∗∗*p* < 0.001, and ∗∗∗∗*p* < 0.0001 were considered significant, and *p* > 0.05 was considered non-significant (ns). The statistical analysis was performed using GraphPad Prism Software.

#### Calling of differentially-methylated regions (DMRs)

DMRs were identified using a segmentation approach.^9^^,^^72^ Coverage profiles were built at single-base resolution using featureCounts^70^ and a log2-FC per-base transformation was applied. The resulting matrix was used as input for a change-point detection algorithm^73^ which identifies segment boundaries in which the log2-FC signal is stable. featureCounts was used again to assign to each segment the mapped reads from each replicate of each biological condition. The resulting matrix was then used to perform differential analysis using DESeq2.^71^ The segments with an adjusted *p*-value *p* < 0.05 and a |log2-FC| > 0.58 were identified as differentially methylated regions (DMRs) and retained for downstream analysis.

#### Quantification of active-seq signal and heatmap generation

Cell type-specific markers were obtained from a human DNA methylation atlas^9^ for the following cell types: Blood-Granul, Breast-Luminal-Ep, Colon-Ep, Liver-Hep, Lung-Ep-Alveo, and Gastric-Ep. Using the corresponding genomic regions, a count matrix was generated with featureCounts from Active-seq samples derived from bulk tissues of blood, stomach, colon, rectum, liver, breast, and lung. Raw counts were normalized by library size to correct for sequencing depth. The matrix was row-scaled using *Z* score normalisation to standardise each feature across samples and facilitate comparison of relative signal intensities. The heatmap was generated using the R package ComplexHeatmap.

#### Genome-wide Active-Seq correlation analysis

Genome-wide correlation between samples was assessed using deepTools (version 3.5.5). In particular, the plotCorrelation function was applied to compute pairwise Spearman correlation coefficients across all samples based on normalised signal values. The results were visualised as a clustered heatmap using hierarchical clustering to assess sample similarity.

## Supplemental information

Document S1. Figures S1–S8 and Tables S1–S3

Data S1. Excel file containing genomic coordinates of background regions and isolated CpG sites, related to Figure S4, and sequencing QC data and patient metadata, related to Figures 6 and 7

Document S2. Article plus supplemental information
