# Supplementary material for: Genome-wide profiling of unmodified DNA using methyltransferase-directed tagging and enrichment
Source: Cell Rep Methods. 2025 Sep 29;5(10):101187. doi: 10.1016/j.crmeth.2025.101187 (PMC12570316; doi:10.1016/j.crmeth.2025.101187)
Supplement: Document S1. Figures S1–S8 and Tables S1–S3 [file mmc1.pdf]

Cell Reports Methods, Volume *5*

Supplemental information

Genome-wide profiling of unmodified DNA using methyltransferase-directed tagging and enrichment

Luca Tosti, Calum Mould, Imogen Gatehouse, Anthony C. Smith, Krystian Ubych, Valentina Miano, Peter W. Laird, Jack Kennefick, and Robert K. Neely

	Ladder	Controls		Cofactor dilution, *R*-adenosyl-L-methionine analogue				Cofactor dilution, *S*-adenosyl-L-methionine analogue				Gel cropped to remove lanes showing activity of M.MpeI with a second, unrelated cofactor	Control -M.MpeI	Control -cofactor	QC lane, enzyme after storage	Control AdoMet + M.MpeI	Ladder
				500 µM	250 µM	125 µM	67.5 µM	500 µM	250 µM	125 µM	67.5 µM						
R.HpaII	_	_	+	+	+	+	+	+	+	+	+		+	+	+	+	_
M.MpeI	_	_	_	+	+	+	+	+	+	+	+		_	+	+	+	_
Cofactor analogue	–	–	–	+	+	+	+	+	+	+	+		+	_	+	+	_
Lane	1	2	3	4	5	6	7	8	9	10	11		16	17	18	19	20

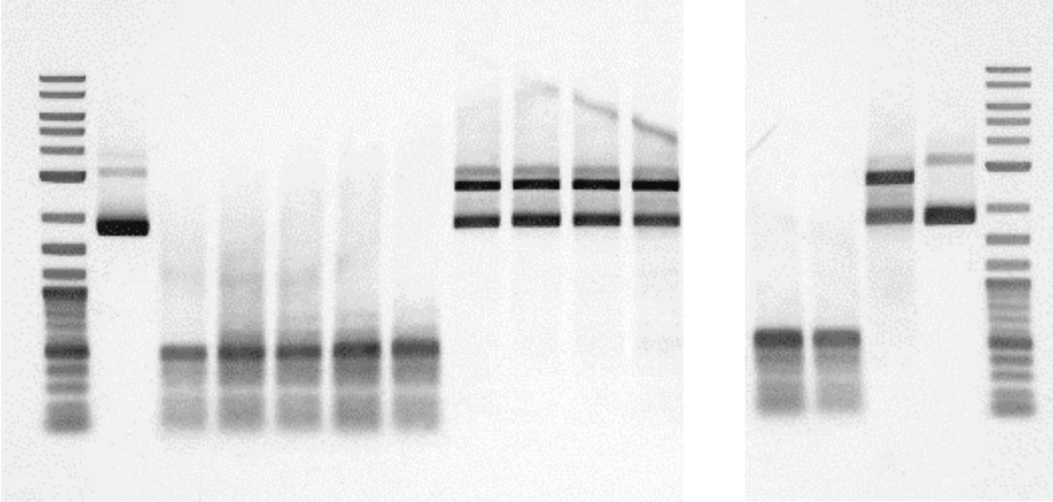

Figure S1, related to Figure 1 and STAR Methods: Agarose gel showing activity of the M.MpeI enzyme on puC19 plasmid DNA, which was challenged with a restriction enzyme (R.HpaII) sensitive to CpG methylation. Lane 2 shows the input DNA, lane 3 is unmodified, completely digested DNA. Two cofactor diastereomers were tested with M.MpeI, which shows negligible activity with (likely) the *R*-adenosyl-L-methionine analogue (lanes 3-7) but near-complete protection of the DNA at cofactor analogue concentrations as low as 68 µM using the *S*-adenosyl-L-methionine analogue (lanes 8-11). Controls show complete digestion of the DNA in the absence of either M.MpeI (lane 16) or the cofactor analogue (lane 17) and complete DNA methylation by M.MpeI in the presence of the native cofactor, *S*-adenosyl-L-methionine (lanes 18-19).

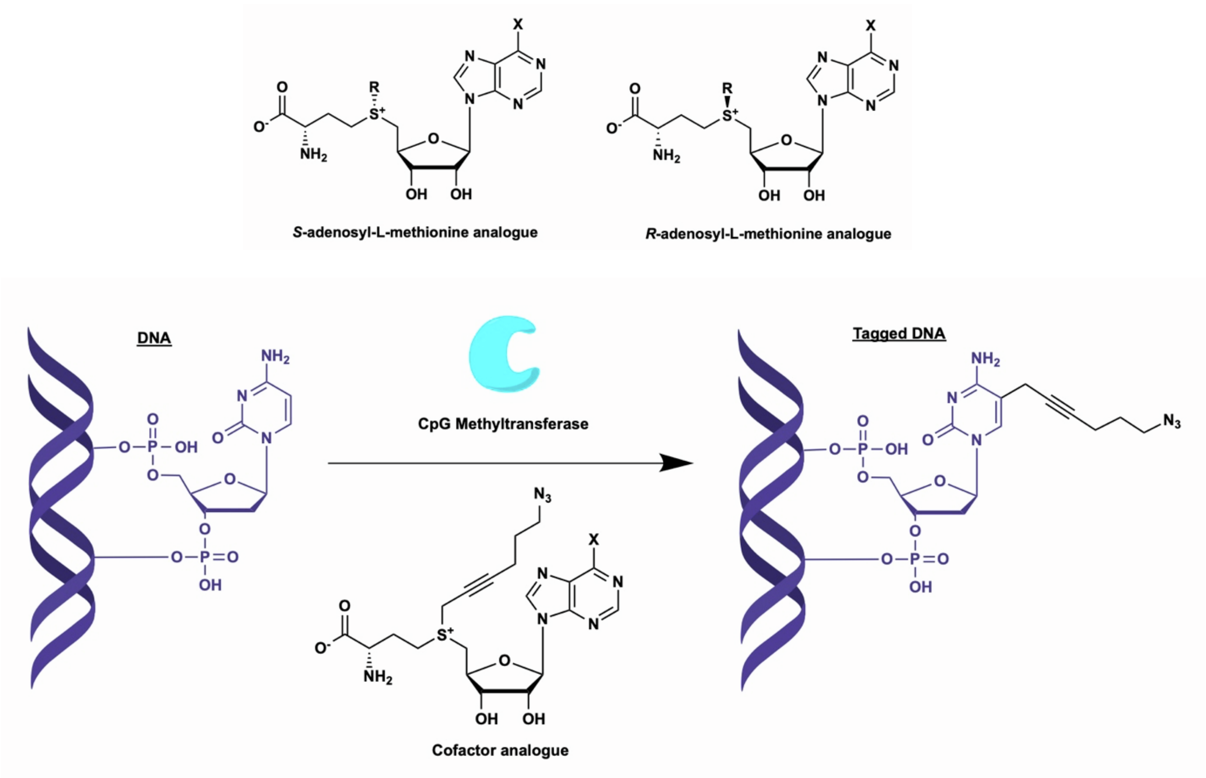

Figure S2, related to Figure 1 and STAR Methods: Chemical structures of the cofactor analogue used for DNA alkylation in this work and the DNA modification introduced during DNA tagging.

Table S1, related to Figure 1B and STAR Methods: Sequences for the primers and PCR products (for the lambda bacteriophage genome (GenBank: J02459.1)) used to produce the short oligonucleotides with defined numbers of CpG sites used for method validation.

Oligo name (number of CpG sites)	DNA sequences
**SEQ_0CG**	GAATCAATTCCAATTACCTGAAGTCTTTCATCTATAATTGGCATTGTATGTAT<br>TGGTTTATTGGAGTAGATGCTTGCTTTTCTGAGCCATAGCTCTGATATCCAA<br>ATGAAGCCATAGGCATTTGTTATTTTGGCTCTGTCAG
**0CG_FWD**	GAATCAATTCCAATTACCTGAAG
**0CG_REV**	CTGACAGAGCCAAAATAAC
**SEQ_1CG**	GCAGGAAGTATGTGGTTACATCAAAACAATTCCCATACATTAGTGAGTTGAT<br>TGAGCTTGGTGTGTTGAACAAAACTTTTTCCCGATGGAATGGAAAGCATATA<br>TTATTCCCTATTGAGGATATTTACTGGACTGAATTAGTTGCCAG
**1CG_FWD**	GCAGGAAGTATGTGGTTAC
**1CG_REV**	CTGGCAACTAATTCAGTCC
**SEQ_2CG**	CATTTCAGGGAGTTGACTGAATTTTTTATCTATTAATGAATAAGTGCTTACTT<br>CTTCTTTTTGACCTACAAAACCAATTTTAACATTTCCGATATCGCATTTTTCAC<br>CATGCTCATCAAAGACAGTAAGATAAAACATTGTAACAAAGGAATAGTCATT<br>CCAACCATCTGCTC
**2CG_FWD**	CATTTCAGGGAGTTGACTG
**2CG_REV**	GAGCAGATGGTTGGAATG
**SEQ_4CG**	CACAACAATGAGTGGCAGATATAGCCTGGTGGTTCAGGCGGCGCATTTTTA<br>TTGCTGTGTTGCGCTGTAATTCTTCTATTTCTGATGCTGAATCAATGATGTCT<br>GCCATCTTTCATTAATCCCTGAACTGTTGGTTAATACGCTTGAGG
**4CG_FWD**	CACAACAATGAGTGGCAG
**4CG_REV**	CCTCAAGCGTATTAACCAAC
**SEQ_10CG**	GCCATTCTGCTTATCAGGAAAGGCGTAAATTTCTTTCGTCCACGGATTAAGG<br>CCGTACTGGTTGGCAACGATCAGTAATGCGATGAACTGCGCATCGCTGGCA<br>TCACCTTTAAATGCCGTCTGGCGAAGAGTGGTGATCAGTTCCTGTGGGTC
**10CG_FWD**	GCCATTCTGCTTATCAGG
**10CG_REV**	GACCCACAGGAACTGATC

Table S2, related to Figure 1D: Sequencing read counts for the plot shown in Figure 1D. The false positive rate derived from these spike-in controls is less than 1% across all spike-in concentrations.

Spike-in %	0.01			0.05			0.1		
Replicate	1	2	3	1	2	3	1	2	3
Total reads in experiment	111040394	102888190	113147438	109576226	105474886	100488712	108801798	101284082	103907034
Total spike-in reads	7688	7313	7661	35652	33924	36639	70863	71617	70783
4 uMe CpG' reads	7581	7218	7574	35131	33477	36201	69945	70700	69993
4Me CpG' reads	71	72	53	351	284	278	584	638	540
0 CpG' reads	36	23	34	170	163	160	334	279	250

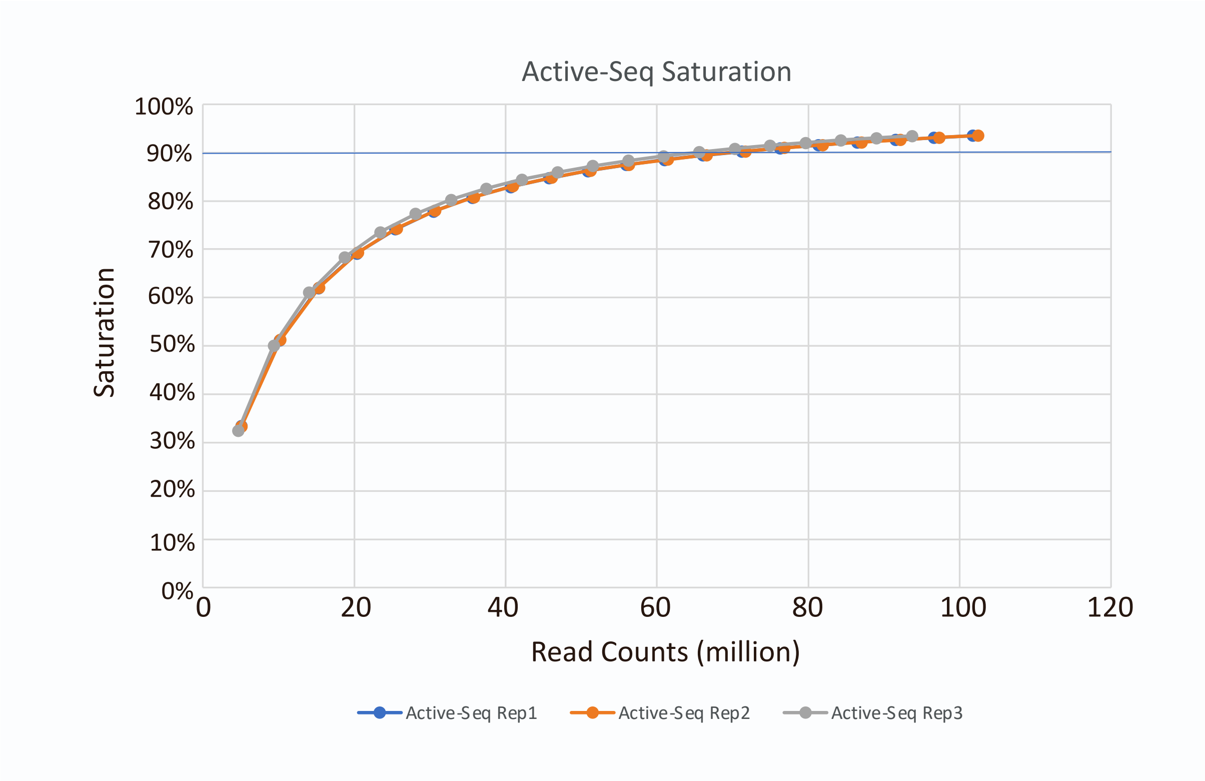

Figure S3, related to Figure 2 and STAR Methods: Sequencing saturation curves generated for three Active-Seq replicates using the R package MEDIPS (1). Experiments consistently reach approximately 90% saturation at 70M reads (150 bp paired-end reads).

Table S3, related to STAR Methods: Output from the Active-Seq MultiQC report, as a function of DNA input to the workflow. Sequencing metrics are consistent across a range of inputs, down to 1 ng. MultiQC summarizes outputs from Qualimap (%GC, genome coverage), SamTools (mapped reads, total reads) and FastQC (duplication rate).

Mass Input DNA	% Background	% GC	Fraction of genome covered			Median coverage	Mean coverage	% Duplicates	% Mapped	M Total reads
			≥ 5X	≥ 10X	≥30X					
1 ng	1.12	47%	32.1%	15.9%	1.0%	2.0X	4.5X	3.9%	100.0%	105.9
1 ng	1.06	46%	21.0%	7.2%	0.1%	1.0X	2.7X	2.7%	100.0%	63.0
1 ng	0.99	47%	31.9%	15.7%	1.0%	2.0X	4.5X	3.9%	100.0%	103.9
10 ng	1.14	47%	36.5%	20.4%	2.2%	2.0X	5.6X	4.3%	100.0%	130.8
10 ng	1.18	46%	37.4%	21.3%	2.5%	2.0X	5.8X	4.4%	100.0%	136.6
10 ng	1.11	47%	38.1%	22.0%	2.8%	2.0X	6.0X	4.5%	100.0%	141.0
50 ng	1.59	46%	33.2%	17.1%	1.3%	2.0X	4.8X	3.8%	100.0%	112.7
50 ng	5.5	46%	38.1%	19.1%	1.4%	3.0X	5.4X	3.9%	100.0%	126.7
50 ng	1.5	47%	37.6%	21.2%	2.4%	2.0X	5.8X	4.5%	100.0%	135.3

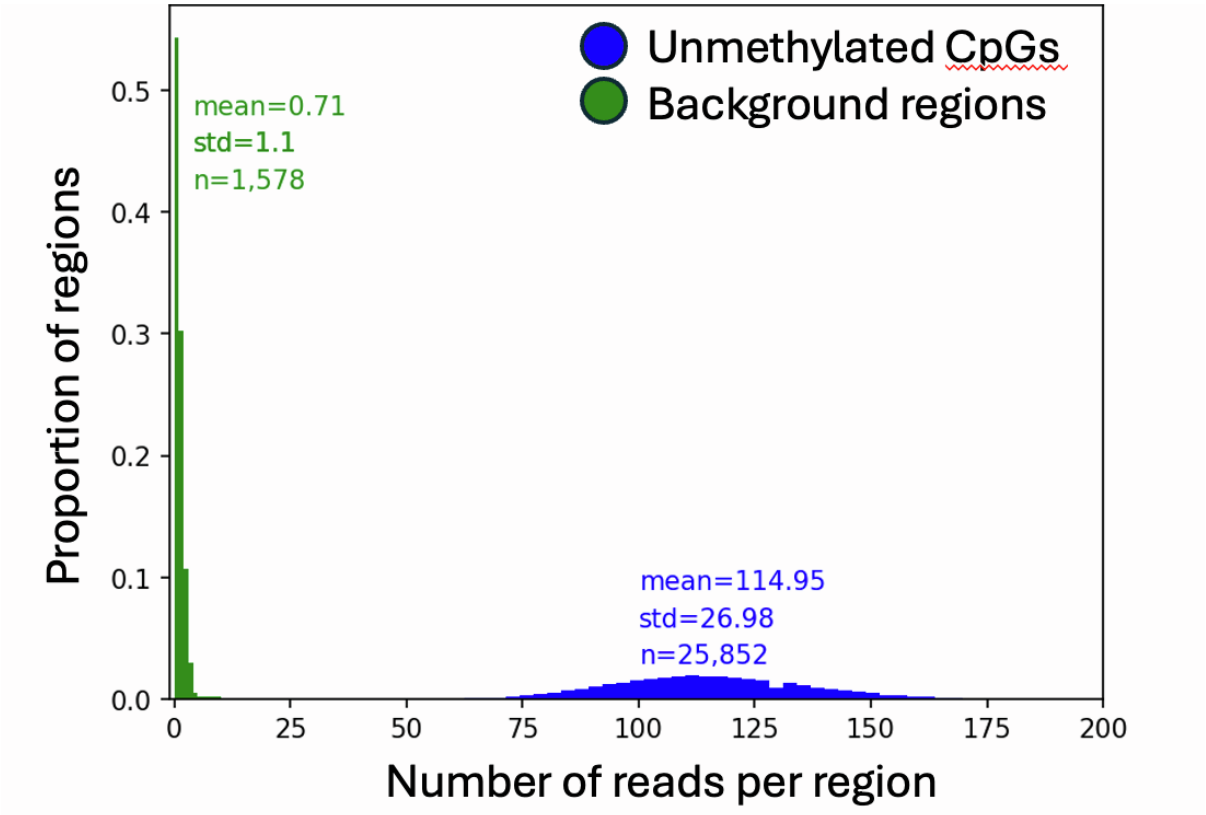

Figure S4, related to Figure 3 and STAR Methods: Enrichment of DNA over background at single, isolated and unmethylated CpG sites. Enrichment is calculated as the ratio of the mean signal in regions containing a single, isolated CpG site (114.95) to the mean signal in regions containing no CpG sites (0.71). An isolated CpG site is defined as a CpG site with a β-value of zero and no other CpG site present within 250 bp up/downstream. This derives a list of 25,852 CpG sites, genome wide. Background regions are regions of the genome of at least 1000 bp in length, containing no CpG sites.

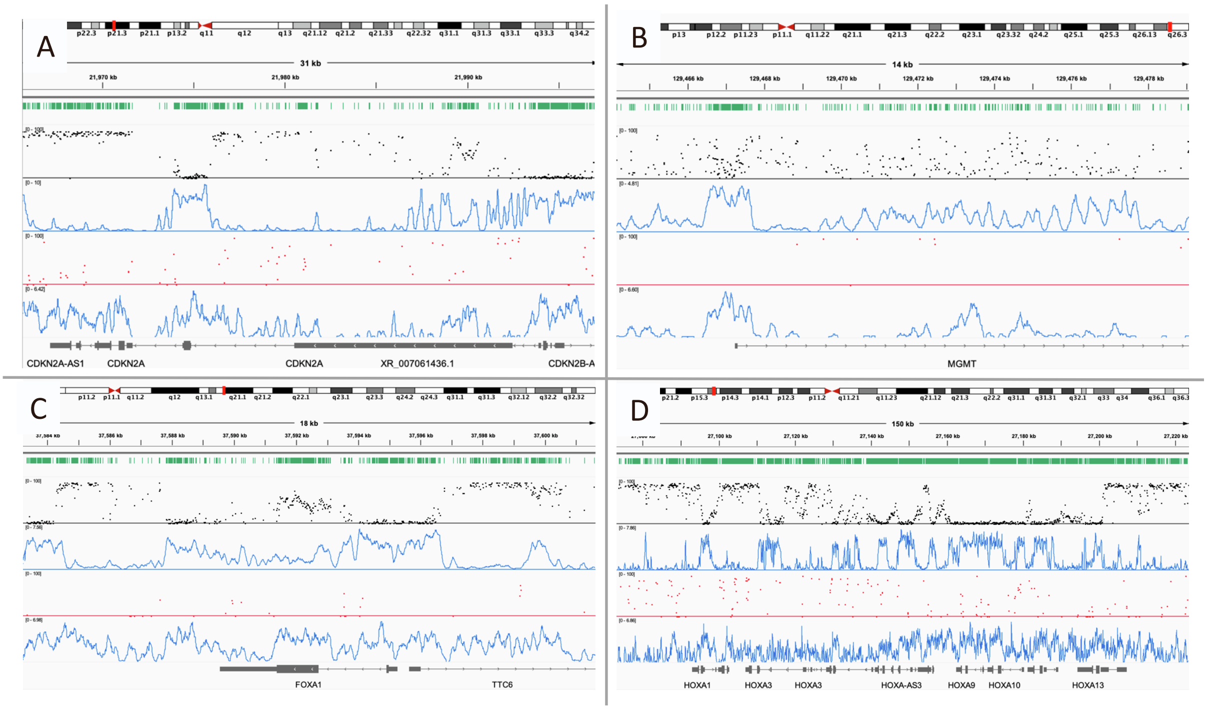

Figure S5, related to Figure 3: Additional screenshots from the IGV genome browser showing the Active-Seq signal mapping against whole genome bisulfite sequencing data for HEK293 (750M reads) (3) and NA12878 (650M reads) (4).

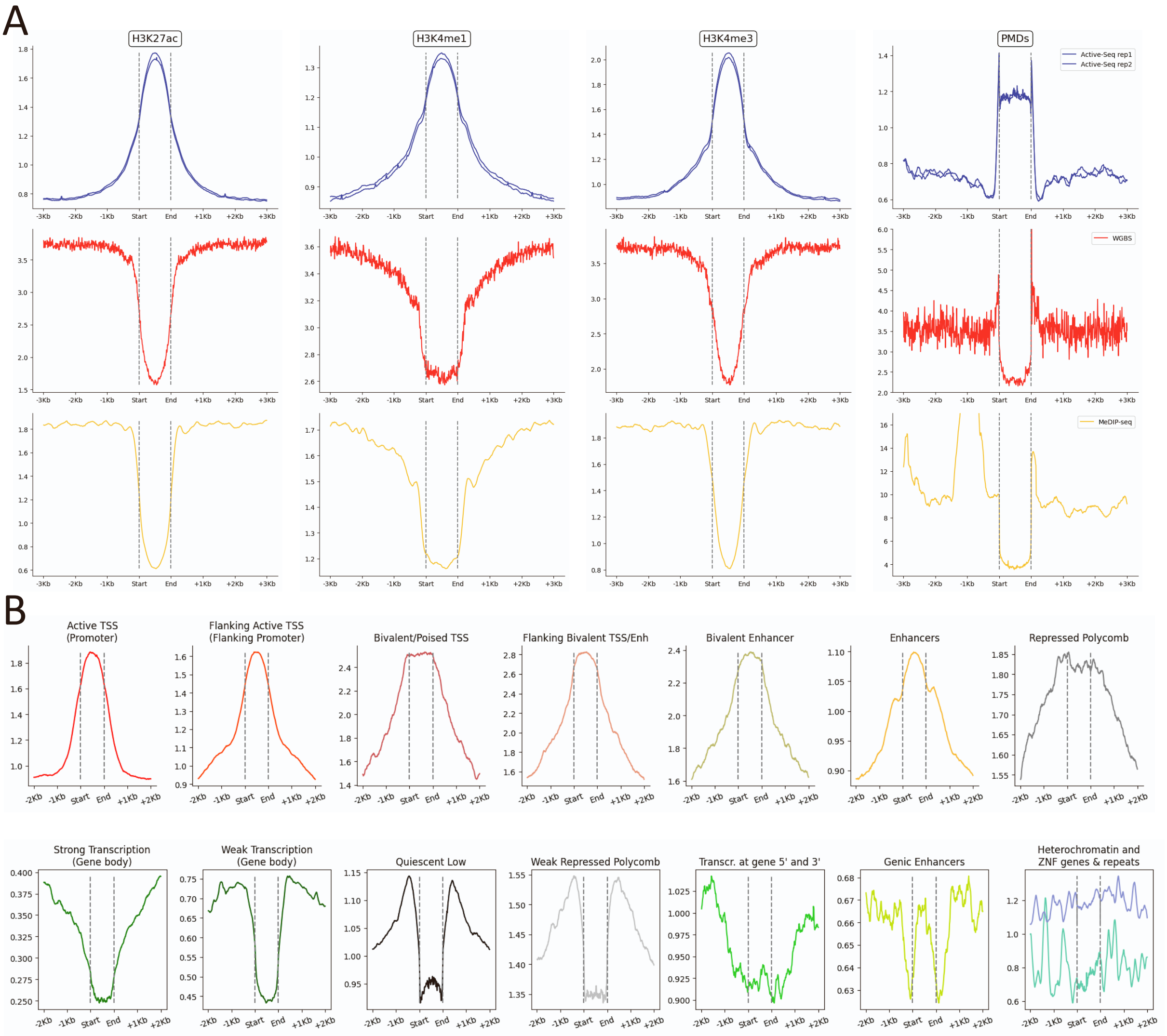

Figure S6, related to Figure 2: Characterisation of Active-seq signal: (**A**) Profile plot of Active-Seq (blue), MeDIP-seq (yellow) and WGBS (red) at genomic regions associated with histone modification marks (H3K27ac, H3K4me1, H3K4me3) and at partially methylated domains (PMDs) in NA12878 DNA. **(B)** Active-Seq profile plots in genomic features previously identified in the NA12878 genome (2).

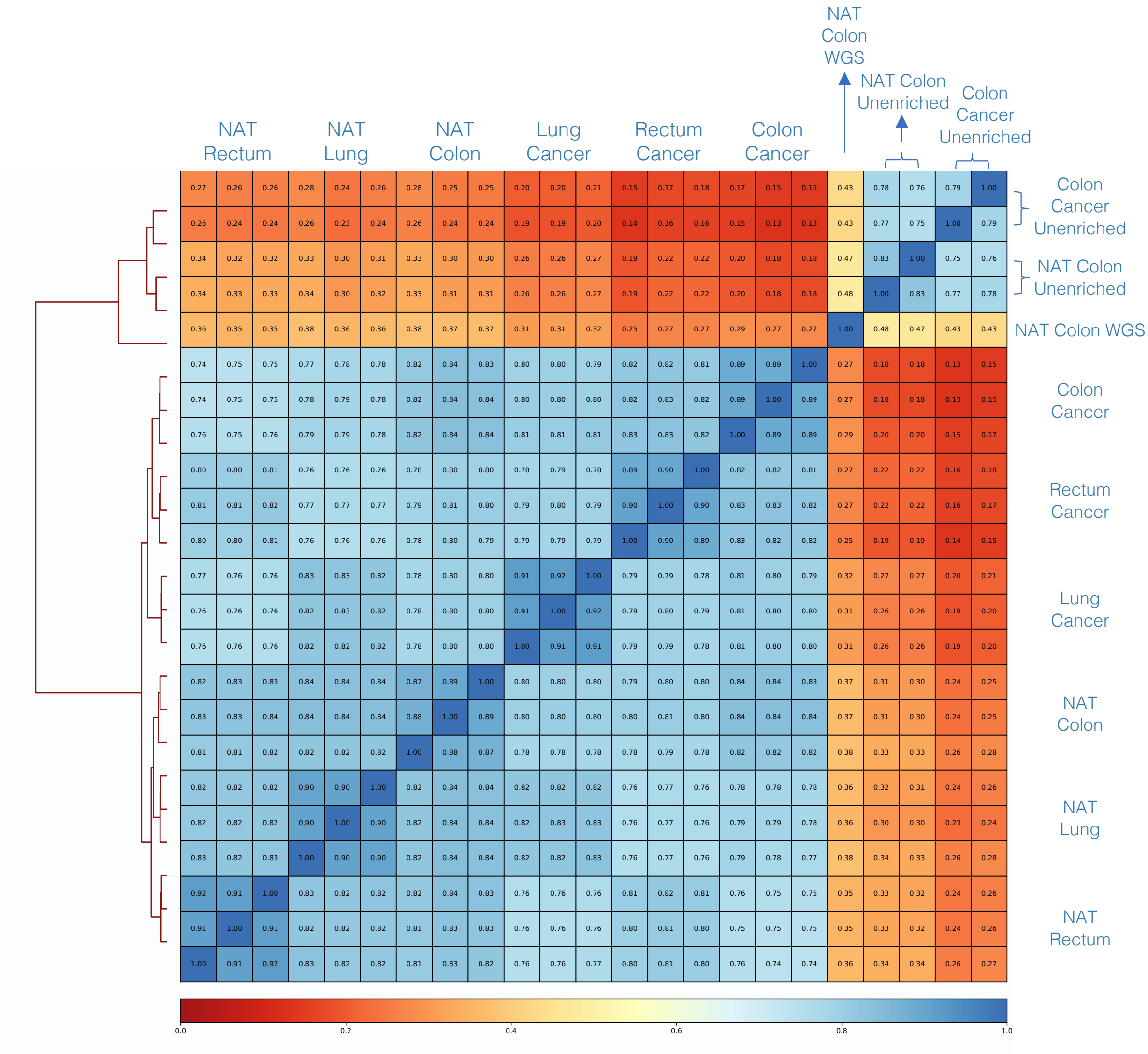

Figure S7, related to Figures 5: Spearman correlation matrix for triplicate repeats of Active-Seq taken from tumour and normal adjacent tissue for patients with colon, rectal and lung cancer. Also shown are the correlations to the unenriched fractions of the genome for colon cancer and to whole genome sequencing for colon cancer.

Patient	Input DNA/ ng	TNM stage	Age	Sex	Diagnosis	Stage
1	7.2	T3N0	58	F	Colon Cancer	2
2	10	T2N0	63	F	Colon Cancer	1
3	1.6	T3N0	57	F	Colon Cancer	2
4	10	T3N1bM1a	60	F	Sigmoid Cancer	4
5	10	T4bN1c	53	M	Adenocarcinoma	3
6	3.8	-	63	F	-	-
7	3.8	-	53	M	-	-
8	3.4	-	48	M	-	-
9	3.8	-	76	F	-	-
10	10	-	47	M	-	-

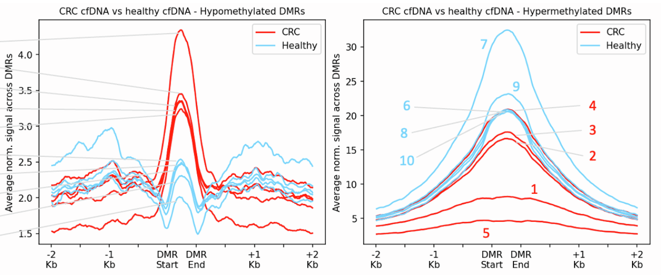

Figure S8, related to Figure 7: Aggregate signal in the cfDNA sample over the hyper- and hypo-methylated DMRs identified in colorectal cancer tissue samples for ten patient cfDNA samples. Same data/patients as in the main text (Figure 7) but with additional information showing the input DNA amounts and TNM staging for the patients.

References

1. Lienhard M, Grimm C, Morkel M, Herwig R, Chavez L. MEDIPS: genome-wide differential coverage analysis of sequencing data derived from DNA enrichment experiments. Bioinformatics. 2014 Jan 15;30(2):284–6.

2. Ernst J, Kellis M. Chromatin-state discovery and genome annotation with ChromHMM. Nat Protoc. 2017 Dec;12(12):2478–92.

3. Zhao S, Lu J, Pan B, Fan H, Byrum SD, Xu C, et al. TNRC18 engages H3K9me3 to mediate silencing of endogenous retrotransposons. Nature. 2023 Nov;623(7987):633–42.

4. Vaisvila R, Ponnaluri VKC, Sun Z, Langhorst BW, Saleh L, Guan S, et al. Enzymatic methyl sequencing detects DNA methylation at single-base resolution from picograms of DNA. Genome Res. 2021 Jul;31(7):1280–9.
